# Onametostat, a PfPRMT5 inhibitor, exhibits antimalarial activity to *Plasmodium falciparum*

**DOI:** 10.1128/aac.00176-24

**Published:** 2024-08-28

**Authors:** Hui Min, Amuza Byaruhanga Lucky, Jesper J. Madsen, Anongruk Chim-Ong, Xiaolian Li, Liwang Cui, Jun Miao

**Affiliations:** 1 Department of Internal Medicine, Morsani College of Medicine, University of South Florida, Tampa, Florida, USA; 2 Department of Immunology, College of Basic Medical Sciences, China Medical University, Shenyang, China; 3 Center for Global Health and Infectious Diseases Research, College of Public Health, University of South Florida, Tampa, Florida, USA; 4 Department of Molecular Medicine, Morsani College of Medicine, University of South Florida, Tampa, Florida, USA; The Children's Hospital of Philadelphia, Philadelphia, Pennsylvania, USA

**Keywords:** *Plasmodium falciparum*, PRMT, antimalarials, arginine methylation

## Abstract

Protein arginine methyltransferases (PRMTs) play critical roles in *Plasmodium falciparum*, a protozoan causing the deadliest form of malaria, making them potential targets for novel antimalarial drugs. Here, we screened 11 novel PRMT inhibitors against *P. falciparum* asexual growth and found that onametostat, an inhibitor for type II PRMTs, exhibited strong antimalarial activity with a half-maximal inhibitory concentration (IC_50_) value of 1.69 ± 0.04 µM. *In vitro* methyltransferase activities of purified PfPRMT5 were inhibited by onametostat, and a shift of IC_50_ to onametostat was found in the *PfPRTM5* disruptant parasite line, indicating that PfPRTM5 is the primary target of onametostat. Consistent with the function of PfPRMT5 in mediating symmetric dimethylation of histone H3R2 (H3R2me2s) and in regulating invasion-related genes, onametostat treatment led to the reduction of H3R2me2s level in *P. falciparum* and caused the defects on the parasite’s invasion of red blood cells. This study provides a starting point for identifying specific PRMT inhibitors with the potential to serve as novel antimalarial drugs.

## INTRODUCTION

According to the World Health Organization, despite substantial progress in reducing malaria infections and fatalities over recent decades, the disease remains one of the most pressing global health challenges, with an estimated 249 million cases and 608,000 deaths in 2022 (1). Nowadays, artemisinin-based combination therapies (ACTs) are widely adopted as the frontline antimalarial drugs. However, the emergence of drug resistance to ACTs threatens the progress made in malaria control, emphasizing the necessity for novel classes of antimalarials that can be used in combination therapies (2, 3). These innovative drugs may be discovered by screening compound libraries against cultured parasites or isolated target molecules validated as essential for parasite viability (4–6). Importantly, to delay or prevent the development of drug resistance, the potential compound should ideally target novel biological activities in the parasite. Recently, extensive high-throughput phenotypic screening has been conducted for the development of antimalarial drugs (7–10).

Epigenetic regulation is a crucial mechanism that contributes to the regulation of gene expression. This process involves remodeling the chromatin structure between active and silent states through various mechanisms, including reversible histone modifications (11). Arginine methylation is a post-translational modification that affects many cellular processes in eukaryotes and plays an important role in many cellular processes, such as RNA processing, signal transduction, and transcription (12, 13). Arginine methylation is catalyzed by a family of protein arginine methyltransferases (PRMTs), which can add one or two methyl groups from S-adenosylmethionine (SAM) to the guanidine–nitrogen of arginine, resulting in three major forms of methylated arginines: MMA–*ω-N*^G^-monomethylarginine, aDMA–asymmetric *ω-N*^G^,*N*^G^-dimethylarginine, or sDMA–symmetric *ω-N*^G^, *N*^G^-dimethylarginine) (13). Types I and II PRMTs catalyze the formation of aDMA and sDMA, respectively. PRMT1 and PRMT5, as the main type I and II enzymes, generate repressive histone markers (H3R8me2s and H4R3me2s) as well as active markers (H4R3me2a and H3R2me2s), respectively (13–15). In addition, PRMTs also deposit arginine methylation on non-histone substrates, regulating essential cellular processes such as transcription, cell signaling, mRNA translation, and pre-mRNA splicing (16). PRMTs play key roles in RNA splicing, cell differentiation, and development and link to diseases such as cancer (17–19). Recently, arginine methylation has attracted increasing attention as a key post-translational modification (PTM), which can be utilized as a therapy for neurodegenerative diseases and cancer (13, 20–22).

*Plasmodium* spp. rely heavily on epigenetic mechanisms to drive both asexual proliferation and sexual differentiation (23–25). *Plasmodium* histones carry a myriad of PTMs, such as methylation, acetylation, phosphorylation, ubiquitylation, and SUMOylation (26–32). The malaria parasite *Plasmodium falciparum* encodes three conserved PRMTs, namely, PRMT1, PRMT4 (also known as CARM1), and PRMT5 (33–35). Our previous studies showed that PfPRMT1 is a type I PRMT located in the nucleus and cytoplasm with activities toward histones and non-histone substrates (34). Recently, PfPRMT5, a type II PRMT, was found to play a critical role in RNA splicing and regulating the invasion of red blood cells (RBCs), partially due to its activation of invasion genes by creating H3R2me2s in the corresponding promoters (36). Therefore, PfPRMTs are promising targets for antimalarials.

Epigenetic inhibitors hold promise as antimalarials, with several studies involving a limited number of such inhibitors demonstrating activity against one or more stages of malaria parasites (37–39). However, the impact of PRMT inhibitors on malaria parasites has not been evaluated yet. In the present study, we screened 11 commercially available PRMT inhibitors to determine their potential antimalarial activity. We found that onametostat, a type II PRMT inhibitor, had the strongest antimalarial activity among the tested compounds. Furthermore, onametostat inhibited PfPRMT5 methyltransferase activities and blocked parasite growth by reducing the level of H3R2me2s and impairing the parasite’s invasion capacity. Additionally, the inhibitory activity of onametostat was attenuated upon *PfPRMT5* gene disruption.

## RESULTS

### Screening PRMT inhibitors in *P. falciparum*

**Fig 1:**
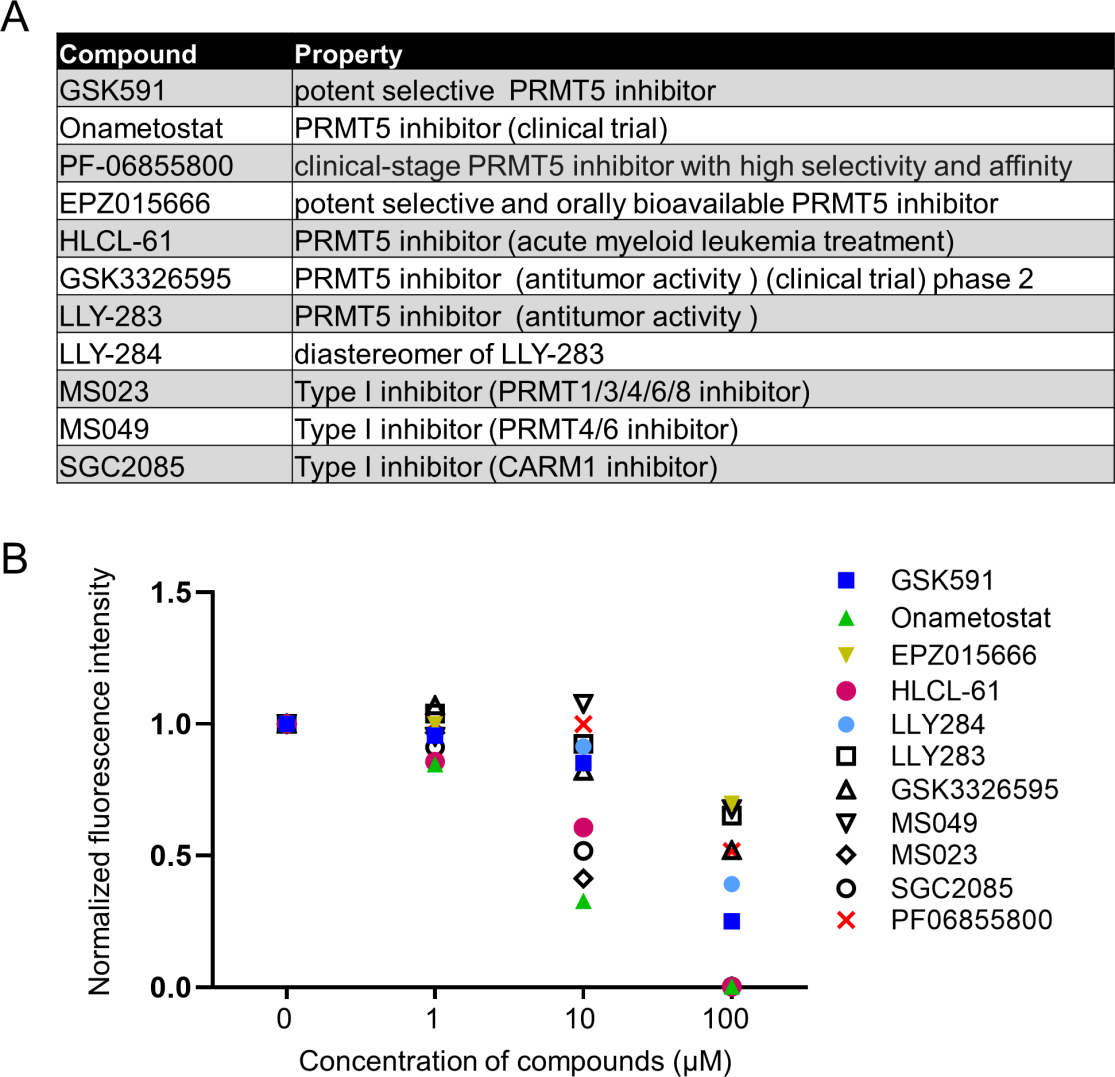
Screening 11 PRMT5 inhibitors in *P. falciparum.* (**A**) Eleven commercially available PRMT inhibitors were selected. Among them, eight compounds are potent PRMT5 inhibitor candidates. (**B**) All the compounds were screened for *in vitro* activity against asexual *P. falciparum* parasites at 0, 1, 10, and 100 µM, respectively.

Based on the studies of PRMT inhibitors against enzymatic activities in the cell-free system, cancer cell lines, or animal models, we selected three type I PRMT inhibitors (MS023 [40], MS049 [41], and SGC2085 [42]) and eight type II PRMT inhibitors (GSK591 [43], GSK-3326595 [44], onametostat [45], PF-06855800 [46], EPZ015666 [47], HLCL-61 [48], LLY-283, and LLY-284 [49]), which showed highly potent inhibitory activities (Fig. 1A). Among them, GSK-3326595, onametostat, and PF-06855800 are being assessed in clinical trials (46, 50).

To assess the activity of the PRMT inhibitors against *P. falciparum*, we performed *in vitro* growth inhibition assays against asexual-stage parasites at 0, 1, 10, and 100 µM of each compound. At 10 µM, MS023 and onametostat inhibited parasite growth by over 50%, whereas SGC2085 and HLCL-61 showed near 50% growth inhibitory activities (Fig. 1B). At 100 µM, these four compounds almost completely inhibited parasite growth, with the fluorescence intensity approaching zero (Fig. 1B).

**Fig 2:**
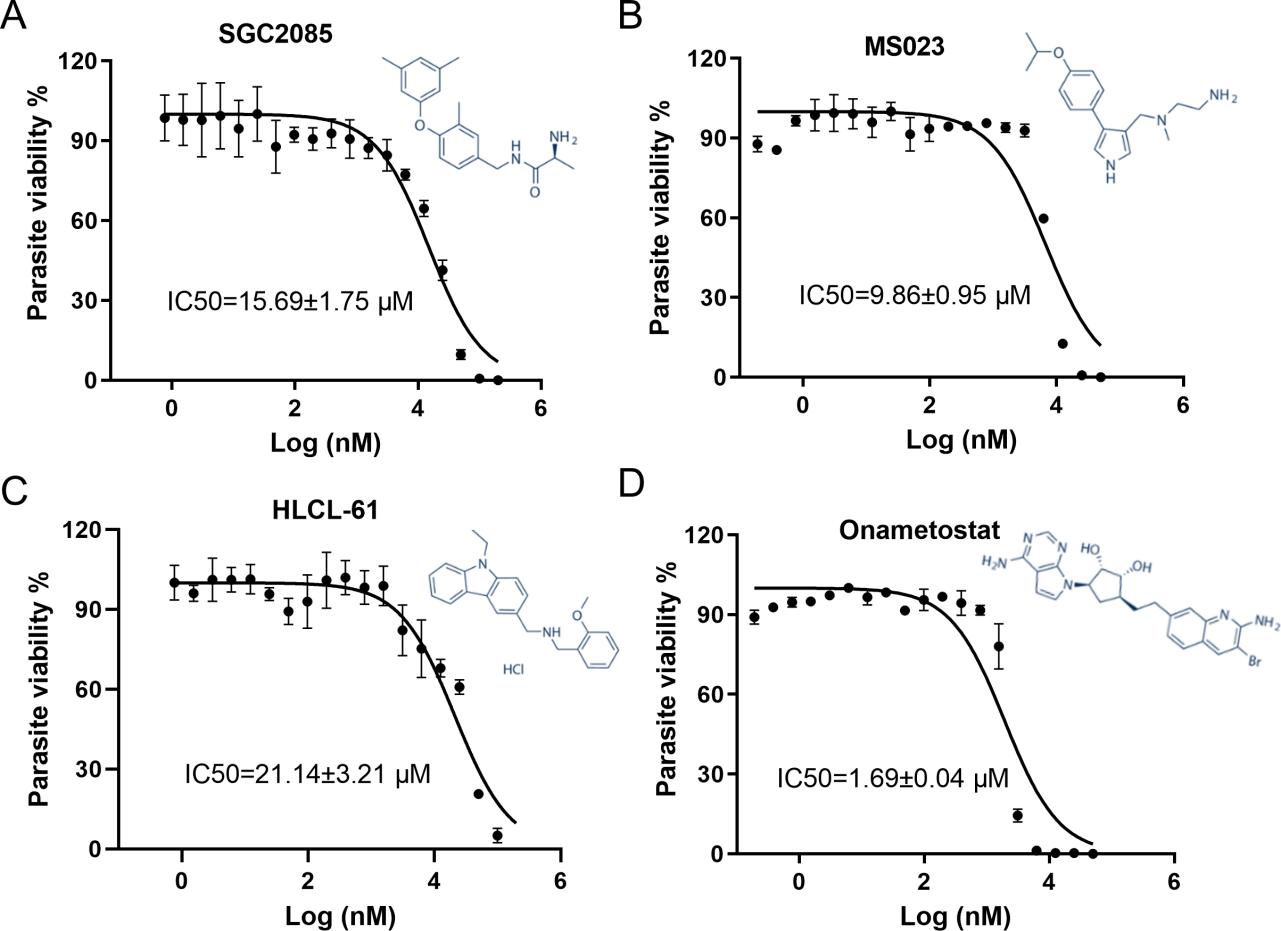
Inhibition curves of PRMT inhibitors against *P. falciparum*. The asexual growth inhibition assay results for SGC2085 (**A**), MS023 (**B**), HLCL-61 (**C**), and onametostat (**D**) are presented as IC_50_ values. The chemical structures of compounds are shown on the right panel.

To further validate the activities of MS023, SGC2085, onametostat, and HLCL-61, we determined their IC_50_ values against parasite strain 3D7 using the *in vitro* SYBR green-based drug assay (Fig. 2). HLCL-61 and SGC2085 showed weak inhibitory activities with IC_50_s at 21.14 ± 3.21 and 15.69 ± 1.75 µM, respectively. MS023 demonstrated a modest antimalarial activity with an IC_50_ value of 9.86 ± 0.95 µM. Onametostat exhibited the strongest activity, with an IC_50_ value of 1.69 ± 0.04 µM. Thus, from the 11 PRMT inhibitors, onametostat was identified as the most potent compound against asexual growth of the malaria parasite.

### Modeling onametostat binding to PfPRMT5

**Fig 3:**
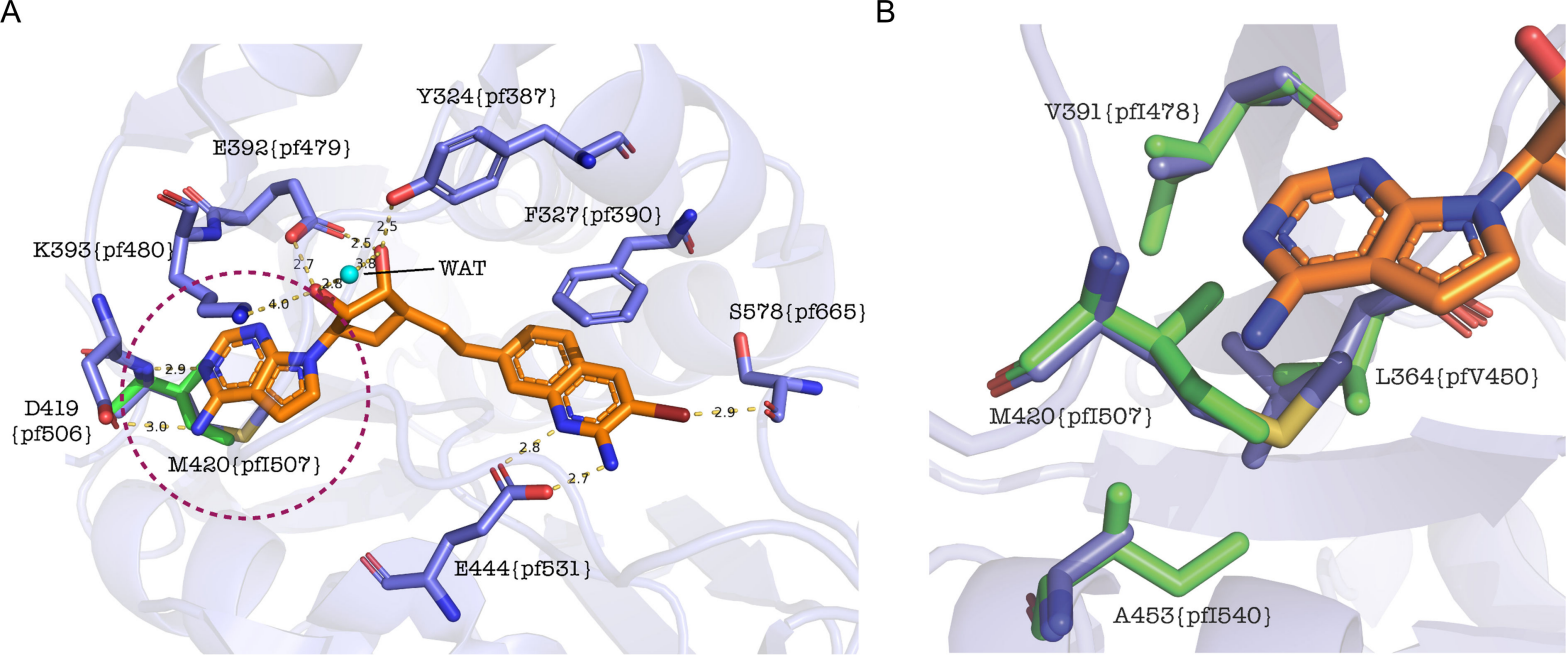
*In silico* modeling shows the binding of onametostat to PfPRMT5. Structural interactions between HsPRMT5 and PfPRMT5 with onametostat are shown. Onametostat is in the stick form (orange). The protein backbone is represented with a ribbon diagram, and crucial amino acids directly interacting with onametostat are illustrated as sticks (blue for Hs; green for Pf). Distances of significance are denoted by dashed lines (yellow), while a coordinating water molecule is visualized as a sphere (cyan). Residue nomenclature adheres to Hs, with the corresponding position in Pf denoted in curly brackets. (**A**) General interaction mode between onametostat and HsPRMT5, with the sole distinction in PfPRMT5’s interaction highlighted within the dashed circle region. This discrepancy involves the substitution of M420 to I507. (**B**) A magnified perspective of the distinct region and the adjacent hydrophobic environment.

The IC_50_s of onametostat in six lung cancer cell lines were at a low nanomolar range (0.4–1.9 nM), significantly lower than the IC_50_ of onametostat in *P. falciparum* (45). Sequence alignment shows that the PfPRMT5 catalytic domain displays high sequence identity/similarity to HsPRMT5 (44/64%) (36). Recently, the co-crystal structure of onametostat bound to the active site of HsPRMT5 (PDB ID: 6RLQ) has been solved, showing that onametostat closely binds several amino acids in the active site (45) (Fig. 3A). Interestingly, almost all respective amino acids are conserved in PfPRMT5 except for M420 in HsPRMT5, which is I507 in PfPRMT5 (Fig. S1).

To fully appreciate this conservation and variation, we explored the differences in onametostat interaction with HsPRMT5 and PfPRMT5 by modeling the known structure of onametostat interaction with HsPRMT5. In the crystal structure of onametostat bound to HsPRMT5, the bromo-aminoquinoline moiety occupies the substrate-binding site, establishing bidentate hydrogen-bonding interactions with the catalytic E444 (E531 in PfPRMT5) (Fig. 3A). Additionally, the bromine atom of onametostat engages in a halogen interaction with the backbone oxygen atom of S578 (S665 in PfPRMT5), and F327 (F390 in PfPRMT5) forms a π–π stacking interaction with onametostat, capping this region of the inhibitor (Fig. 3A). The central dihydroxycyclopentane moiety of onametostat forms bidentate hydrogen bonds with E392 (E479 in PfPRMT5) and a hydrogen bond with Y324 (Y387 in PfPRMT5), while also coordinating with a water molecule (Fig. 3A). Although the distance to K393 (K480 in PfPRMT5) is relatively large at 4.0 Å, limiting its interaction with the central group, this residue engages in a cation–π interaction with the pyrrolopyrimidine moiety of onametostat. Notably, the interaction with the enzyme around the pyrrolopyrimidine group involves a hydrogen bond to the backbone nitrogen atom of M420 (I507 in PfPRMT5) (Fig. 3A). However, differences arise in the interaction between onametostat and HsPRMT5 versus PfPRMT5, particularly concerning the hydrophobic environment surrounding the pyrrolopyrimidine group. This interaction involves M420/I507, which contributes to the hydrophobic milieu where the pyrrolopyrimidine moiety nests. Structural analysis reveals differences in this hydrophobic neighborhood around M420/I507, including substitutions of V391 by I478, L364 by V450, and A453 by I540 (Fig. 3B). These differences likely contribute to distinct affinity profiles, offering a potential avenue for further exploration and optimization.

### Specific targeting of PfPRMT5 by onametostat

**Fig 4:**
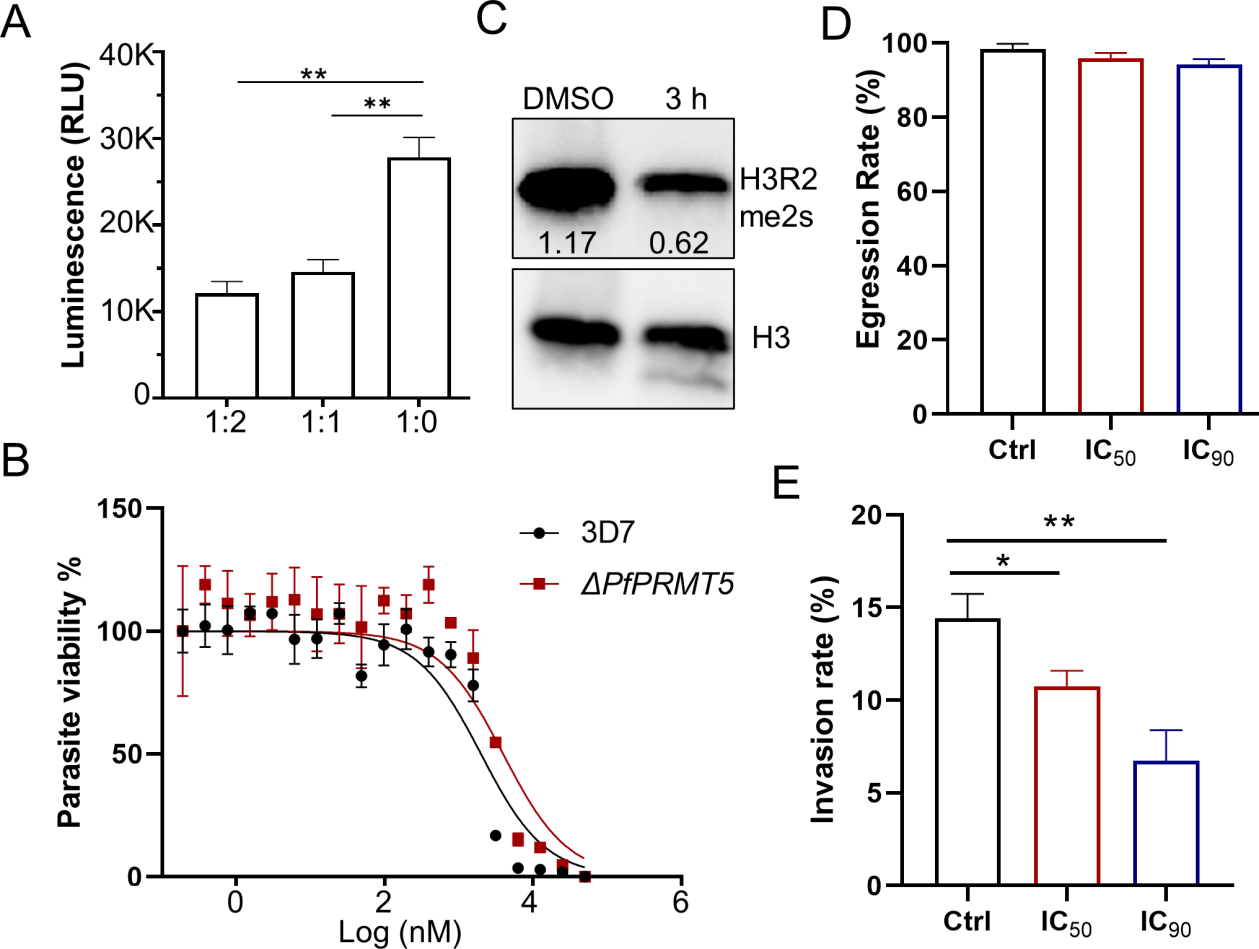
The effects of onametostat on *P. falciparum*. (**A**) The activity of the purified PfPRMT5 was significantly inhibited by onametostat at a molar ratio of 1:1 and 1:2 of PRMT5 to onametostat. The activities were measured using *in vitro* methyltransferase activity assays and shown as relative light units (RLU). (**B**) Comparison of the *in vitro* susceptibility of *ΔPfPRMT5* parasite line with 3D7 exposed to onametostat. (**C**) The level of H3R2me2s was detected by Western blot after parasites at the schizont stage were treated with either dimethyl sulfoxide (DMSO) or onametostat at IC_50_ for 3 h. H3 was used as a loading control. The numbers show the relative levels of H3R2me2s normalized with H3. (**D**) Onametostat at IC_50_ and IC_90_ did not significantly disturb schizont egress. The egress rate was defined as the ratio of naturally ruptured mature schizonts from three replicates. (**E**) Onametostat at IC_50_ and IC_90_ concentrations substantially disturbed merozoite invasion. Invasion rates of parasites were estimated using purified schizonts. Error bars in all panels indicate the mean ± SD from three replicates. *<*I>P* < 0.05, **<*I>P* < 0.01.

To experimentally confirm that onametostat can specifically inhibit PfPRMT5, we investigated the inhibitory effects of onametostat on PfPRMT5 methyltransferase activities. We first purified PfPRMT5 from the PfPRMT5::PTP parasite line by tandem affinity purification (TAP) procedure as shown in our previous pulldown (36, 51) (Fig. S2A). The purified PfPRMT5 was functional and showed high activities by *in vitro* methyltransferase assay (Fig. S2B). Adding onametostat into the reaction mixture at 1:1 and 1:2 molar ratio of PfPRMT5 to onametostat resulted in ~48% and ~66% reduction of its activities, respectively (Fig. 4A; Fig. S2C), indicating that onametostat could directly inhibit PfPTMT5 activity. Disruption of target genes will dampen the inhibitory effects of the respective drugs (6, 52). Therefore, we anticipated that disruption of *Pf*PRMT5 would lessen onametostat’s inhibitory activity. As expected, the IC_50_ of onametostat in the *PfPRMT5* disruptant line was increased >2-fold (3.88 ± 1.61 µM) compared to the wild-type parasite, whereas the IC_50_ of onametostat in an unrelated gene disruption line, *PfDNMT2* disruption line (53), did not change compared to its wild-type control (Fig. 4B; Fig. S2D).

Our recent studies showed that PfPRMT5 regulated gene expression by depositing the active histone marker, H3R2me2s, in the promoters of target genes, including certain invasion genes at the schizont stage, and disruption of *PfPRMT5* resulted in the reduction of H3R2me2s and defects in the invasion of RBCs (36). To investigate whether onametostat targets PfPRMT5, onametostat at the IC_50_ concentration was added to highly synchronized schizont-stage parasites for 3 h. Compared to the DMSO control, the level of H3R2me2s was significantly reduced following onametostat treatment (Fig. 4C). When highly synchronized ring-stage parasites were incubated with onametostat at the IC_50_ concentration, no dead parasites were detected during the intraerythrocytic developmental cycle (Fig. S3). The egress process of schizonts was normal under onametostat treatment at either IC_50_ or IC_90_ (Fig. 4D), suggesting that onametostat did not block the parasite development and might instead impair the merozoite invasion process. Therefore, we quantified the invasion rates and found that onametostat treatment at IC_50_ or IC_90_ significantly reduced the parasite’s invasion rate (Fig. 4E). Collectively, these results showed that onametostat impaired the parasite asexual stage growth by specifically targeting PfPRMT5.

## DISCUSSION

This study investigated 11 inhibitors known to target mammalian PRMTs for their antimalarial activities against the asexual stages of *P. falciparum*. We showed that the selective PRMT5 inhibitor, onametostat, exhibited significant anti-proliferative efficacy against asexual *P. falciparum* parasites and specifically inhibited type II PRMT (PfPRMT5) activities.

Many compounds potentially targeting epigenetic modulators have been proven active against cancerous cells (54, 55). Notably, PRMT5 inhibitors have shown promising results in clinical trials for treating certain types of tumors (56–58). A growing body of literature has demonstrated that epigenetic regulation is becoming an attractive molecular target in the design of novel antimalarials (38, 59). Recently, epigenetics drugs were screened for activities against the asexual or sexual stages of cultured *P. falciparum*, including histone deacetylases, histone lysine methyltransferases, and PRMT inhibitors, but no lead compounds were identified (37, 39). In this study, four of the 11 selected PRMT inhibitors inhibited parasite proliferation by ~50% at 10 µM, with onametostat displaying the most potent activity at an IC_50_ value of 1.69 µM.

Onametostat inhibits PRMT5 activity by binding non-covalently in the SAM binding site and substrate-binding pockets(45). It is a highly selective PRMT5 inhibitor, with a high binding affinity to PRMT5 and a Kd of ≤1 nmol/L in human lung cancer cell lines. The catalytic domain of PfPRMT5 exhibits 44% sequence identity and 64% sequence similarity to HsPRMT5/JBP1 (36). Notably, among the amino acids in HsPRMT5 directly interacting with onametostat, only one is not conserved in PfPRMT5 (Fig. S1). Detailed structural analysis suggested that onametostat specifically targeted the catalytic domain of PfPRMT5 but with relatively lower inhibition activity against *Plasmodium* parasites compared to cancer cells.

Our recent study showed that PfPRMT5 catalyzes the formation of symmetrical methylation of H3R2 to mediate transcriptional activation in *P. falciparum* (36). H3R2me2s was enriched in the promoters of certain genes, including invasion genes, and disruption of *PfPRMT5* resulted in defects in merozoite invasion of RBCs. Furthermore, PfPRMT5 is also associated with invasion-related transcriptional regulators, such as AP2-I and RNA splicing machinery, which play key roles in regulating splicing of many RNA species including those encoding invasion-related genes (36). In this study, onametostat could inhibit PfPRMT5 activity by *in vitro* methyltransferase assay. Additionally, onametostat specifically reduced the level of H3R2m2s and invasion capacity in the wild-type parasite, and the PfPRMT5 disruptant line became less sensitive to onametostat. All these demonstrate that onametostat is a specific inhibitor of PfPRMT5. It is anticipated that onametostat also disturbs RNA splicing by inhibition of PfPRMT5 in *P. falciparum*.

Besides onametostat, the other three PRMT5 inhibitors tested in this study, namely MS023, SGC2085, and HLCL-61, inhibited parasite growth at a low micrometer range (~10–20 µM), underscoring the significance of PfPRMTs as the promising drug targets. Further detailed analyses of drug–PRMT interactions and inhibition phenotypes are needed to facilitate rational drug design and optimization.

## MATERIALS AND METHODS

### Parasite culture

The *P. falciparum* parasites were cultured in type O^+^ human RBCs at 5% hematocrit in RPMI 1640 medium supplemented with 25 mM HEPES, 50 mg/L of hypoxanthine, 25 mM NaHCO_3_, 0.5% Albumax II, and 40 mg/mL of gentamicin sulfate. The deidentified and anonymous human blood was purchased from BioIVT. The culture was maintained at 37°C in a gas mixture of 5% CO_2_, 3% O_2_, and 92% N_2_. The *∆PfPRMT5* parasite line was generated earlier (36). For synchronization, the culture was initiated with purified parasites using a Percoll step gradient, and the ring-stage parasites were treated with 5% sorbitol.

### *In vitro* drug sensitivity assay

The *in vitro* susceptibilities of the wild-type and mutant parasites to 11 PRMT inhibitors were tested using the SYBR green I-based assay (60). Briefly, synchronized ring-stage parasites were cultured with each selected compound at 1% hematocrit and 0.5% parasitemia in a 96-well plate. Each compound at each concentration was applied in two wells (two technical replicates). Wells with no drug and only RBCs were used as a positive control and the background, respectively. After 72 h of incubation, the cells in the plates were frozen, thawed, and then lysed using 100 µL of lysis buffer (20 mM Tris-HCl, [pH 7.5], 5 mM EDTA, 0.08% Triton X-100, 0.008% saponin in phosphate buffered saline [PBS], and 0.2 µL of SYBR green I). The plates were incubated at 37°C for 1 h in the dark after thorough mixing, and fluorescence intensities in each well were measured using a spark multimode microplate reader (TECAN). For each drug, the array was performed three times independently at three separate time points. The values from these three replicates were used for calculating the percentage of parasite growth against the control after subtracting the RBC background signal. IC_50_ and IC_90_ values were calculated using Prism 8 (GraphPad Software Inc.) by fitting a curve with nonlinear regression (sigmoidal dose–response/variable slope equation).

The PRMT5 inhibitor PF-06855800 was purchased from ChemieTek, while the rest of the PRMT inhibitors were from Selleck Chemicals. The stock solutions were prepared with DMSO to a concentration of 5 or 10 mg/mL. To screen the inhibitors’ effects on *P. falciparum*, each compound was tested at a concentration of 1, 10, or, 100 µM. Parasites treated with the solvent DMSO were used as controls. To determine the IC_50_ of onametostat, HLCL-61, MS023, and SGC2085 synchronized ring-stage 3D7 or *∆PfPRMT5* parasites were cultured with serial dilutions of each drug in a 19-point dose–response gradient (concentration range, 0.019 nM to 50 µM).

### Structural modeling

The structural configuration of HsPRMT5 and its inhibition by onametostat have been elucidated through the co-crystal structure with PDB ID: 6RLQ (45). For comparative analysis, the PfPRMT5 structure was modeled by employing 6RLQ as a template using SWISS-MODEL web server (61). The default parameters and settings of the SWISS-MODEL were used. Additionally, structure prediction for PfPRMT5 was performed using AlphaFold2 (62, 63), to validate the template-based model. With the substantial sequence identity of PfPRMT5 to the template, both the template-based and AlphaFold2-predicted models exhibited minimal structural deviation compared with the HsPRMT5 template (root-mean-square deviation for the methyltransferase domain was 0.146 and 0.424 Å over 132 and 130 alpha-carbon atoms, respectively). The structural models were examined using PyMOL (Version 2.5.0, Schrödinger LLC) for visual inspection and verification.

### Purification of histones and PfPRMT5

Histones were isolated from the schizont stage of synchronized parasites as described previously (36). Parasites were harvested with saponin lysis. The parasite pellets were washed with cold PBS till the supernatant was clear. Then, the pellet was resuspended in a hemoglobin-removal buffer (25 mM Tris–HCl, pH 7.8, 1 mM EDTA, 0.2% Nonidet P40) to remove hemoglobin. After washing with 0.8 M NaCl two times, the histones were extracted with 0.25 M HCl and incubated at 4°C for 3 h. Acid-soluble fractions were pooled and mixed with equal volumes of 20% tri-chloroacetic acid and incubated on ice for 30 min. Histones were precipitated by centrifugation at 12,000 × *g* for 15 min at 4°C, washed with 500 µL of acetone, dried, and dissolved in an SDS-PAGE sample buffer.

PfPRMT5 was purified from the PfPRMT5::PTP parasite line by the established TAP procedure (36, 51). Briefly, ~5  ×  10^8^ synchronized schizonts were lysed in the PA150 buffer. The lysate was centrifuged for 10  min at 16,000  ×  *g*, and the supernatant was incubated with IgG agarose beads (100  µL, GE Healthcare) at 4°C for 2  h. The beads were equilibrated with the TEV buffer. and PfPRMT5-PTP was released from beads by digestion with TEV protease (150 U) overnight at 4  °C. The supernatant was collected and incubated with anti-protein C beads for 2 h at 4°C. The proteins that bound to the beads were eluted with an elution buffer. The final eluate was collected for *in vitro* methyltransferase assay.

### Western blots

An equal volume of the wild-type 3D7 parasites at the schizont stage was treated with either DMSO or onametostat for 3 h. After treatment, parasites were harvested, and their histones were extracted as described above. Protein concentration was measured using the Pierce BCA Protein Assay Kit (Thermo Fisher Scientific). The purified histones were separated by 10% SDS-PAGE and transferred to nitrocellulose membranes. Western blotting was performed using a standard procedure with rabbit anti-histone H3R2 dimethyl symmetric (H3R2me2s) (1:1,000, Epigentek), which was verified by dot blots in our recent PfPRMT5 functional study (36), as the primary antibodies and horseradish peroxidase-conjugated goat anti-rabbit IgG (1:3,000) as the secondary antibodies. Rabbit anti-H3 (1:1,000, Epigentek) was used as the loading control. The results were visualized using the ECL detection system to confirm the PfPRMT5 was pulled down by TAP from PfPRMT5::PTP parasites. The elute from TAP was separated by 10% SDS-PAGE followed by Western blots with anti-protein C antibodies (1:1,000, GenScript).

### *In vitro* methyltransferase assay

Methyltransferase activity of PfPRMT5 and inhibitor assay were performed using the MTase-Glo assay (Cat. No. V7601, Promega) following the manufacturer’s protocol (64). For the enzyme activity assay, serial dilutions of PfPRMT5 ranging from 50 to 0.05  µg or no protein as control were incubated in the presence of 0.1  µM human recombinant mononucleosomes (rNucs) (Cat. No. 16–0009, EpiCypher) in reaction buffer (80  mM Tris pH 8.0, 200  mM NaCl, 4  mM EDTA, 12  mM MgCl_2_, 0.4  mg/mL of BSA, 4  mM dithiothreitol), along with 10 µM S-adenosylmethionine (SAM). The total reaction volume was 20  µL, and incubation was performed for 60 min at 37°C in 96-well plates (Cat. No. 15042, Pierce). After incubation, 5  µl of 5 × MTase Glo Reagent (Promega) was added followed by shaking for 2 min at 1,000  rpm and additional incubation at room temperature for 30 min. Subsequently, 25  µl of MTase-Glo Detection Solution (Promega) was added, and the plates were shaken again for 2 min and incubated for another 30 min at room temperature. Luminescence was measured as relative light units (RLU) using a spark multimode microplate reader (TECAN). For inhibitor activity assay, purified PfPRMT5 was pre-incubated with varying concentrations of onametostat or DMSO as a control for 30  min before adding the remaining components. The assay wells received 17.5 µL of reaction buffer containing 115 µM PfPRMT5 and onametostat at molar ratios of 1:2, 1:1, and 1:0, followed by 30 min of incubation at 37°C. Then, 12.5 L of reaction buffer, containing SAM (10 µM) and rNucs (0.1  µM), along with 5  µL of 5 × MTase Glo Reagent, were added. The reactions were incubated for 60 min at 37°C, and then 30 µL of MTase-Glo Detection Solution was added, followed by a 30-min incubation at room temperature. Luminescence was quantified shortly after addition. Data were expressed as relative luminescence by subtracting the luminescence from a negative control reaction without onametostat.

### *In vitro* evaluation of parasite egress and invasion

To assess the impact of onametostat treatment on parasite egress, highly synchronized mature schizonts were exposed to onametostat at IC_50_ and IC_90_ concentrations. Egress rate was defined as the proportion of mature schizonts that naturally ruptured. The invasion assay was performed as previously described (36). Purified mature schizonts were incubated with RBCs to allow schizont rupture and re-invasion. Ring-infected erythrocytes were counted after Giemsa staining. All the assays are performed in triplicate.

### Statistical analysis

Phenotypic comparisons between 3D7 and *∆PfPRMT5* parasite lines were carried out using GraphPad Prism, version 8.0. The egress and invasion rates in the onametostat treatment groups were compared with the control using one-way analysis of variance (ANOVA). Dunnett’s multiple comparison test was employed for multiple comparisons. Data are represented as mean ± standard deviation (SD) from three independent experiments. Unless indicated otherwise, *P* < 0.05 was taken as statistically significant.

## SUPPLEMENTAL MATERIAL

Supplemental figures aac.00176-24-s0001.pdf Figures S1 to S3.

ASM does not own the copyrights to Supplemental Material that may be linked to, or accessed through, an article. The authors have granted ASM a non-exclusive, world-wide license to publish the Supplemental Material files. Please contact the corresponding author directly for reuse.
