# Supplementary material for: Onametostat, a PfPRMT5 inhibitor, exhibits antimalarial activity to Plasmodium falciparum
Source: Antimicrob Agents Chemother. 2024 Aug 28;68(10):e00176-24. doi: 10.1128/aac.00176-24 (PMC11459956; doi:10.1128/aac.00176-24)
Supplement: Supplemental figures — Figures S1 to S3. [file aac.00176-24-s0001.pdf]

Supplementary Figures

Onametostat, a PfPRMT5 inhibitor, exhibits antimalarial activity to *Plasmodium falciparum*

A

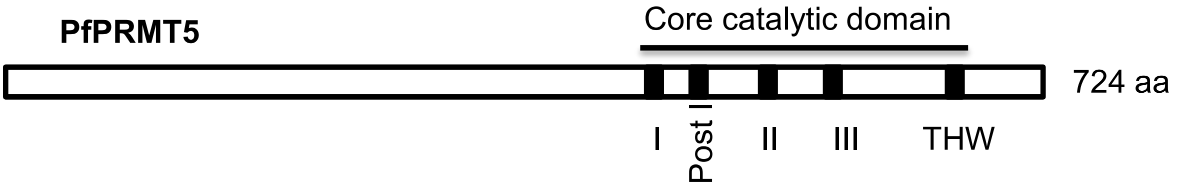

B

```
                #(F327:F390)
TbPRMT5  ASGVYEVFEQDRTKYQQYHTAMSKYFNEWLNHSESRSHEKMWLQGPNPRDG---CGCSAM 428
PfPRMT5  SSQTYEVFEKDRKKYEQYELATSKYLSNWKKGKKINNKNKQSNKNKNNNNNNNMEEQQND 442
HsPRMT5  ESQTYEVFEKDPIKYSQYQQAIYKCLLDRVP-----------------------EEEKDT 356
SpSKB1   ENITYEIFERDPVKYAQYEQAIFSALMDRDE----------------------------S 343
          . .**:**:*  ** **. *  . : :

              I                        Post I
          __________                   ________(E392:479)
TbPRMT5  YVVLLGAGRGPLISECLCAATGV-GVRVHLFVVEKNPEALELVRLRVRADPQWHDWMNYS 490
PfPRMT5  TIFVVGAGRGPLVDTTLSALQKNEMTDYEIYAIEKNDSAIIILNNRVQTE--EWK----- 498
HsPRMT5  VLMVLGAGRGPLVNASLRAAKQA-DRRIKLYAVEKNPNAVVTLENWQFEE--WGS----- 411
SpSKB1   RIAVVGAGRGPLVDCALRAAISS-SRTVDMIALEKNPNAFSMLLMRNRQD--WAG----- 398
          : ::*******:.   * *         .: .:*** .*.  :      :

                                          II   Δ        Δ
              (D419:D506) (M420:I507)  ________        (E444:E531)
TbPRMT5  GHVVETIYADGRSVWSGEAPGSDDRLPPYWGLCDLVVSELLGSFGDNELSPECLDDFYCN 550
PfPRMT5  --NVKVIHSDIRY-L------------DIPKKADIIISELLGSFGDNELFPECMDGIKKF 543
HsPRMT5  --QVTVVSSDMRE-W------------VAPEKADIIVSELLGSFADNELSPECLDGAQHF 456
SpSKB1   --KVTLVFGDMRT-W------------NPDYKIDILVSELLGSMGDNELSPECLDGVQHV 443
            *  : .* *                     *:::******:.**** ***:*.
         ____________________
                III
TbPRMT5  LLSYQESSGIPCNPYLTSIPQQYTAWIAPLHSARMEESVATAAFGGLTTPPADCHDRHAA 610
PfPRMT5  LKD----DG-------ISIPMNCVSYLEPISCSALYHKVMENNI--------------SG 578
HsPRMT5  LKD----DG-------VSIPGEYTSFLAPISSSKLYNEVRACREKDRD-P--------EA 496
SpSKB1   LDE---ETG-------ICIPSSYISYVTPIMSPKLWSEARNMND--------------PN 479
         * .     *        .** .  ::: *: .  :  ..

TbPRMT5  LYHSMFVSNVCR-----AVGLCLPQPCWTFHHFATKV------QSKEREATLNFTLSGDG 659
PfPRMT5  GNESFYVVNMYSYTKISQES---SKECFFFQVPPIHT--KQDNSHNYRYKNINFKIKMDT 633
HsPRMT5  QFEMPYVVRLHNFHQLSAP-----QPCFTFSHPNRDPM-----IDNNRYCTLEFPVEVNT 546
SpSKB1   AFERQYVVLMNSFDFLAADDEFRFQSLWSFHHPNKDSEVYTKNLHNKRFASVRFQASSPG 539
           .  :*  :              :  : *     .         : *  .:.*  .

                                                 THW loop
                                            ______________(S578:S665)
TbPRMT5  RFSGFICYFSAVLFTPGDTGNVEDSIALLCASAGSLSTVQYGRTTGLFSWFPAFLPVEPR 719
PfPRMT5  YIHGFLCYFKSQLYDDVYISIE--------------PK---THTPNLHSWFPLYIPINKI 676
HsPRMT5  VLHGFAGYFETVLYQDITLSIR--------------PE---THSPGMFSWFPILFPIKQP 589
SpSKB1   ILHGFAGYFEATLYKDISLSIM--------------PATMEAKSPDMFSWFPIYMPIKKP 585
          : **   **.: *:     .                      :: .:.****  :*::
```

Supplementary Figure 1. PfPRMT5 is a type II PRMT with conserved methyltransferase domains. (**A**) Schematic representation of PfPRMT5 showing the conserved core catalytic domains (motifs I, Post I, II, III, and THW) and a long non-conserved N-terminus. (**B**) Amino acid sequence alignment of methyltransferase domains among four PRMT5 from *P. falciparum* (PfPRMT5), *Trypanosoma brucei* (TbPRMT5; GenBank Q38CH6), *Homo sapiens* (HsPRMT5; GenBank O14744), *and Saccharomyces pombe* (SpSKB1; GenBank P78963). Asterisks (*) indicate fully conserved residues, colons (:) indicate conservation between groups of strongly similar properties, and periods (.) indicate conservation between groups of weakly similar properties. The AdoMet binding domain (motifs I, Post I, II, and III) and the THW domain are indicated above the sequences. The double-E loop is marked as triangles. The conserved phenylalanine (F) critical for Type II enzyme is marked with a #. The amino acids in HsPRMT5 which binds onametostat are highlighted in yellow and the conservation of these amino acids in PfPRMT5 are highlighted by yellow (conserved) and red (not conserved).

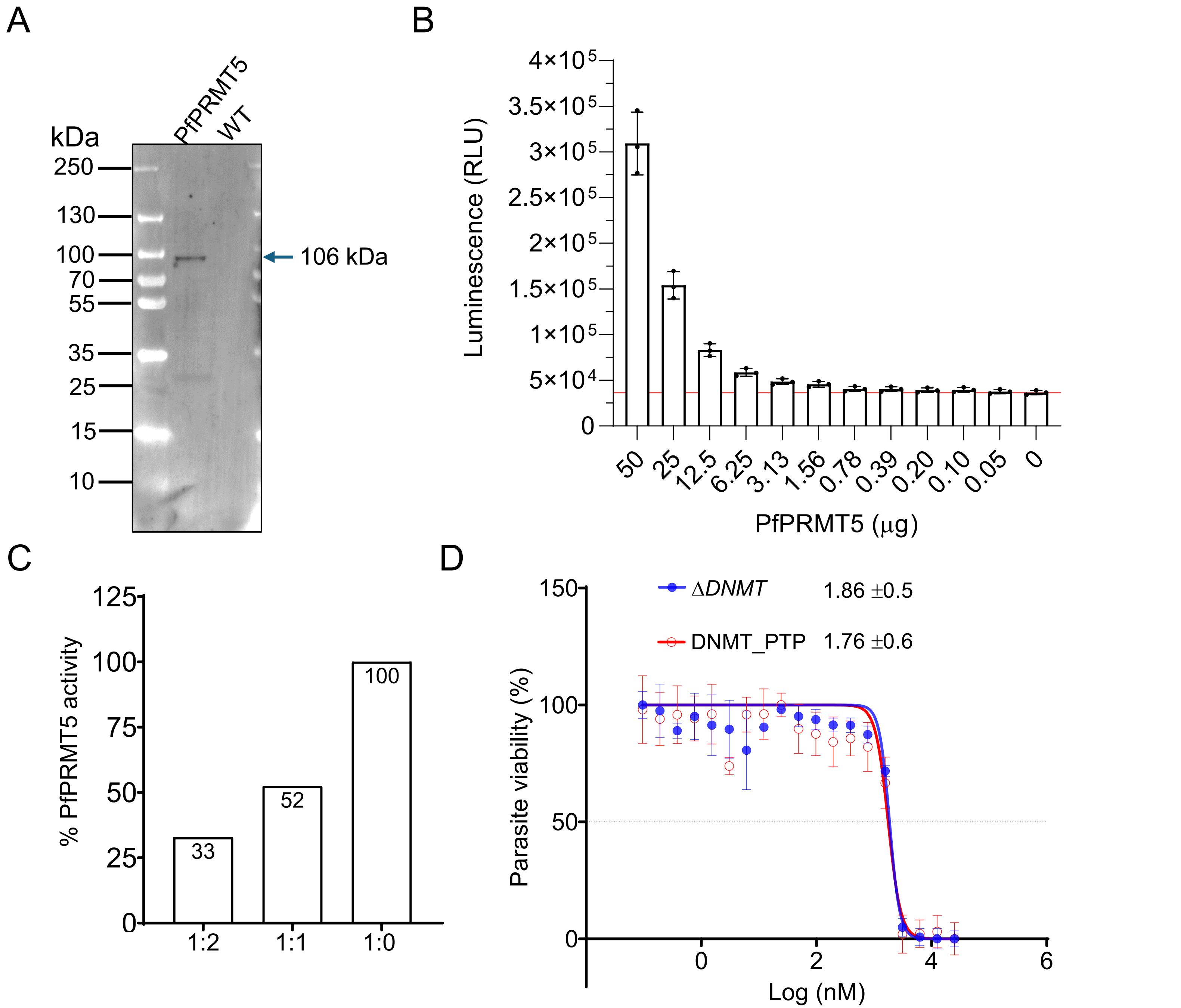

Supplementary Figure 2. Inhibition of PfPRMT5 activity by onametostat. (**A**) PfPRMT5 was purified by tandem affinity purification (TAP) from PfPRMT5::PTP parasite line. Western blot showed the elute of TAP contained the PfPRMT5 proteins compared to the pulldown from wild-type parasites. (**B**) The purified PfPRMT5 had methyltransferase activity by *in vitro* methyltransferase activity assay using MTase-Glo assay kit. RLU: relative light unit. (**C**) The activity of the purified PfPRMT5 was inhibited by onametostat at molar ratio of 1:1 and 1:2 of PRMT5 to onametostat. The activities were measured using *in vitro* methyltransferase activity assays and shown as percentage of activity compared to the PfPRMT5 activity without inhibitor. (**D**) Comparison of the *in vitro* susceptibility of *ΔDNMT* parasite line with its control (DNMT:PTP line) exposed to onametostat.

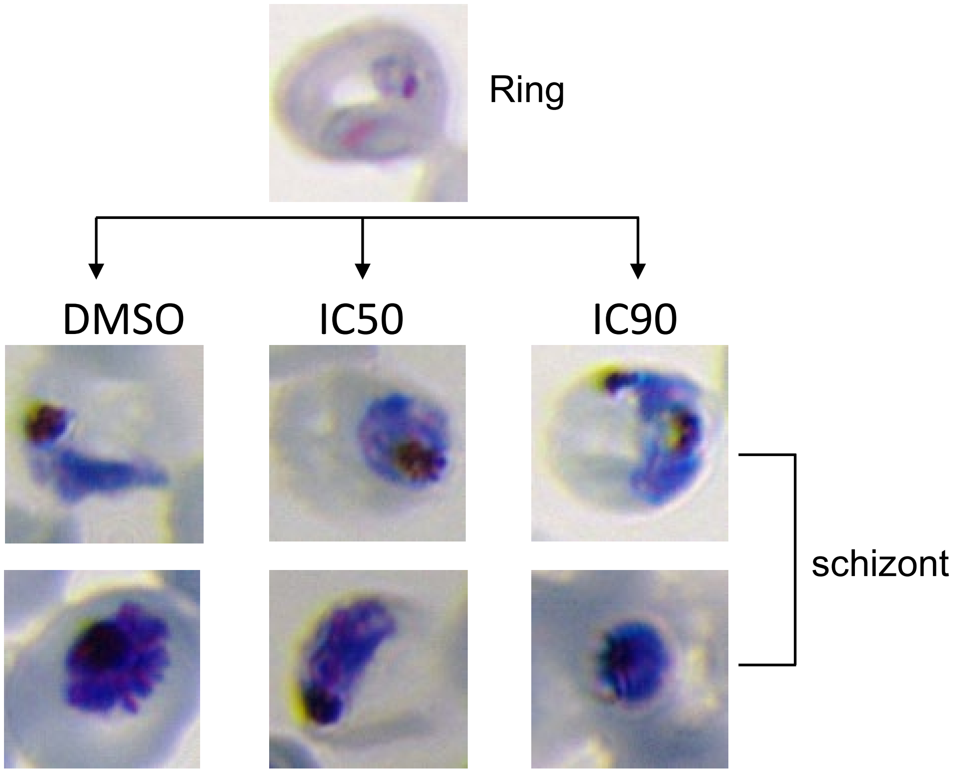

Supplementary Figure 3. onametostat does not inhibit asexual parasite development. 3D7 parasites were incubated with onametostat at IC_{50} and IC_{90} concentrations starting at the ring stage and the parasite growth was monitored by Giemsa staining. The representative schizonts were shown indicating that parasites can develop into mature schizont under onametostat treatment similar to DMSO treatment.
